# Controlled Formation of Porous Cross-Bar Arrays Using Nano-Transfer Printing

**DOI:** 10.3390/ma17225609

**Published:** 2024-11-16

**Authors:** Yu Na Kim, Eun Bin Kang, Tae Wan Park, Woon Ik Park

**Affiliations:** Department of Materials Science and Engineering, Pukyong National University (PKNU), Busan 48513, Republic of Korea; kyn510000@gmail.com (Y.N.K.); beiunn803@gmail.com (E.B.K.); twpark0125@gmail.com (T.W.P.)

**Keywords:** nano-transfer printing, cross-bar pattern, nanoporous, nanostructure

## Abstract

Nano-transfer printing (nTP) has emerged as an effective method for fabricating three-dimensional (3D) nanopatterns on both flat and non-planar substrates. However, most transfer-printed 3D patterns tend to exhibit non-discrete and/or non-porous structures, limiting their application in high-precision nanofabrication. In this study, we introduce a simple and versatile approach to produce highly ordered, porous 3D cross-bar arrays through precise control of the nTP process parameters. By selectively adjusting the polymer solution concentration and spin-coating conditions, we successfully generated discrete, periodic line patterns, which were then stacked at a 90-degree angle to form a porous 3D cross-bar structure. This technique enabled the direct transfer printing of PMMA line patterns with well-defined, square-arrayed holes, without requiring additional deposition of functional materials. This method was applied across diverse substrates, including planar Si wafers, flexible PET, metallic copper foil, and transparent glass, demonstrating its adaptability. These well-defined 3D cross-bar patterns enhance the versatility of nTP and are anticipated to find broad applicability in various nano-to-microscale electronic devices, offering high surface area and structural precision to support enhanced functionality and performance.

## 1. Introduction

Nanopatterning enables the creation of various nanoscale structures, such as nanodots [1,2,3], nanopillars [4,5,6,7], nanospheres [8,9], nanotubes [10,11,12], and nanolines [13,14]. Photolithography is one of the most commonly employed techniques for fabricating these nanostructures due to its precision and scalability in pattern transfer processes [15,16,17,18,19]. However, photolithography has limitations, particularly in patterning on curved surfaces, and forming high-resolution, ultrafine patterns requires costly equipment and complex processing, leading to significant expenses [20]. To overcome these challenges, several alternative nanopatterning techniques have been developed, including nano-transfer printing (nTP) [21,22,23,24,25], nanoimprint lithography (NIL) [26,27,28,29], dip pen (DPN) lithography [30,31], extreme ultraviolet (EUV) lithography [32], and atomic force microscopy (AFM) lithography [33]. Among these, thermally assisted nano-transfer printing (T-nTP) has emerged as an innovative face-to-face patterning approach, offering increased efficiency in nanopatterning processes [34]. In particular, the nTP-based patterning methods enable the fabrication of diverse two-dimensional (2D) and three-dimensional (3D) nanostructures [35,36], with 3D stacking being especially advantageous for maximizing pattern density in limited substrate areas [37,38].

For 3D nanostructure fabrication, block copolymer (BCP) and nano-transfer printing (nTP) processes are frequently utilized. The BCP process is capable of producing mesh-like porous patterns [39,40], with 3D nanostructures formed through the stacking of multiple layers [41]. The nTP process also supports the 3D stacking of patterns, allowing for the creation of intricate 3D patterns on rigid and/or flexible substrates or by stacking mold patterns [42,43,44]. Despite advances in high-resolution 3D patterning based on BCP and large-area 3D structuring using T-nTP, challenges remain in expanding the range of compatible materials and simplifying the process to enhance practicality [45]. In particular, for the nTP process, there is a need to broaden its applicability by developing techniques for creating non-continuous or discrete replica patterns, rather than relying on the complex process of coating functional materials on replica patterns followed by transfer printing [37].

In this work, we introduce a novel method for fabricating 3D cross-bar patterns using poly(methyl methacrylate) (PMMA) by optimizing T-nTP process conditions. We demonstrate the ability to produce discrete patterns by precisely controlling key parameters, including mold design, PMMA concentration, and spin-coating speed. Furthermore, we show that this technique can be applied to various substrates, enabling the formation of 3D PMMA porous arrays on different surfaces and thereby enhancing the versatility of this approach.

## 2. Materials and Methods

### 2.1. Separation of Replication Patterns of Si Master Mold

The Si mold, fabricated through a photolithography process, was designed with a line-to-space ratio of 1:8 and a height of 350 nm. To facilitate the release of PMMA replication patterns from the Si master mold, hydroxyl-terminated polydimethylsiloxane (PDMS-OH, Polymer Source Inc., Dorval, QC, Canada) with a molecular weight (MW) of 5 kg/mol was spin-coated onto the mold at 5000 rpm for 23 s. Following spin-coating, the coated mold was thermally treated at 150 °C for 2 h to ensure adhesion and the stability of the PDMS layer. The CH_3_ groups in the PDMS-OH layer introduce hydrophobic and nonpolar characteristics to the mold surface, enhancing the mold release properties. This treatment minimizes adhesion between the PMMA and the mold, allowing for easy separation of the replicated patterns.

### 2.2. Polymer Solution Preparation

In this study, four polymer solutions of varying concentrations were prepared to regulate the polymer content. PMMA with a MW of 120 kg/mol (Sigma Aldrich Co., St. Louise, MI, USA) was used as the polymeric material, dissolved in a solvent mixture consisting of acetone (99.5%, Junsei Co., Tokyo, Japan), toluene (99.5%, Junsei Co.), and heptane (99.0%, Junsei Co.) in a volumetric ratio of 4:4:2. The resulting PMMA solutions were prepared with concentrations ranging from 2.5 to 4 wt%, with increments of 0.5 wt%. These prepared solutions were employed for the fabrication of polymeric patterns.

### 2.3. Characterization

The transferred PMMA polymer patterns were examined using a field emission scanning electron microscope (FE-SEM, Quattro S, Thermo Fisher Scientific (FEI), Waltham, MA, USA) under an acceleration voltage of 15 kV. The working distance was maintained at 10 mm to optimize imaging resolution and clarity. To achieve high pattern transfer fidelity in the nTP process, we first control and examine the thickness of the PMMA on both the line and space regions of the Si mold after spin-coating. Using FE-SEM, we observe the PMMA patterns both on the Si mold before transfer and on the target substrate after printing, focusing on the width and thickness of the PMMA lines and spaces.

## 3. Results and Discussion

### 3.1. Nano-Transfer Printing for 3D Polymer Multi-Stacking

**Figure 1:**
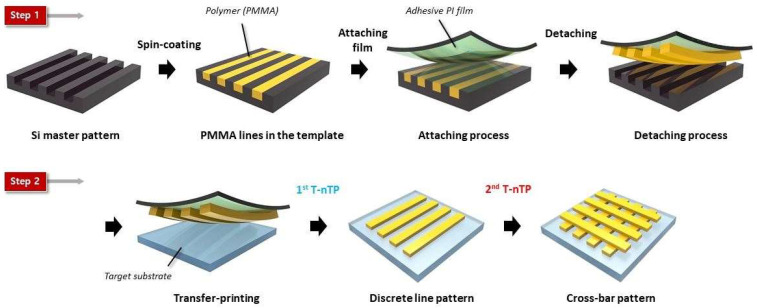
Schematic illustration of the pattern formation process for PMMA cross-bar structures using pattern-transfer printing. Step 1: PMMA pattern replication—the spin-coated PMMA layer is peeled off from the Si master mold using PI tape. Step 2: transfer printing—the discretized PMMA patterns are transferred onto the target substrate.

The process for forming a 3D polymeric multilayer structure is illustrated in Figure 1. The T-nTP process consists of two main steps: (1) PMMA pattern replication and (2) transfer printing of the PMMA replica pattern. In step 1, the PMMA solution is spin-coated onto a photolithographically fabricated Si mold at an appropriate rotational speed and time. To obtain a discrete line pattern, it is crucial to ensure that the PMMA only fills the recessed areas (space regions) of the master mold during spin coating. After spin-coating, the PMMA layer is attached to an adhesive polyimide (PI) tape and then carefully separated from the Si mold. In step 2, the PMMA replica pattern is transferred onto the target substrate using uniform heat and pressure. To achieve the formation of well-defined patterns on various substrates, we employed a hot rolling press system (LAMIART-470 LSI, GMP Corp., Busan, Republic of Korea), which provides consistent heat and pressure. The laminating process was performed at a temperature of 150 °C and a speed of 200 mm/min. After passing through the laminator, the PI tape is removed, leaving the transferred PMMA pattern on the substrate. Under optimal PMMA replication conditions, discrete line patterns can be obtained after transfer printing. By repeating the process at a 90-degree angle relative to the original pattern, a cross-bar structure is formed, creating a 3D polymer multilayer structure.

### 3.2. Control of the Spin-Coated PMMA Thickness on the Si Mold

**Figure 2:**
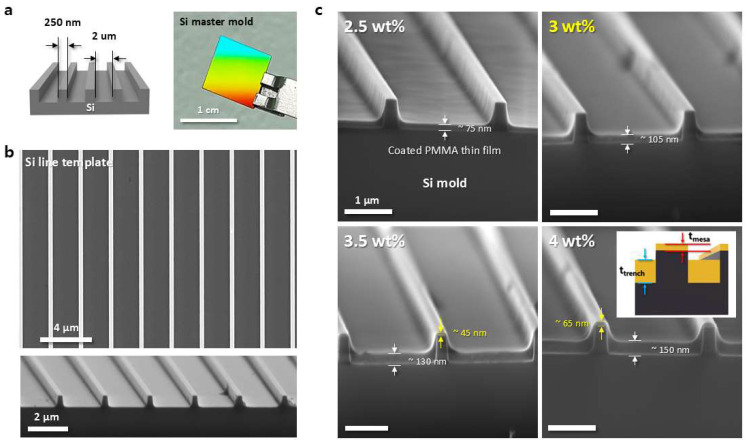
Control of the spin-coated PMMA thickness on the Si mold as a function of PMMA concentration. (**a**) Si master mold with a space width of 2 µm and a line width of 250 nm, fabricated via conventional photolithography. Scale bar: 1 cm. (**b**) SEM image of the Si master mold. Scale bars: 4 µm, 2 µm. (**c**) Filling level of the Si mold as influenced by PMMA concentration. Scale bar: 1 µm. The thickness of the PMMA film increases proportionally with the weight percentage of the PMMA solution.

To achieve a discrete pattern where each line is not connected, the grooves or spaces (trench) in the master mold must be filled with polymer, while the raised areas (mesa) should have minimal to no coating. Figure S1 shows the process sequence for a typical nTP process when using the replica pattern for patterning functional materials, showing a continuous PMMA film with micro-channels. Prior to printing on the target substrate, it is important to examine the effect of the PMMA weight percentage (wt%) in order to obtain the appropriate PMMA film thickness for creating discrete line patterns from the Si master mold. Figure 2 illustrates how the thickness of the spin-coated PMMA film on the Si mold can be controlled as a function of PMMA concentration. Figure 2a provides a schematic and a photograph of the patterned Si master mold. To successfully replicate the polymer pattern, the PMMA solution must adequately fill the space regions of the mold. For this purpose, a mold with a line-to-space ratio of 1:8, favoring the space regions, was used. Generally, the thickness of the PMMA film increases proportionally with the weight percentage of the PMMA solution. Figure 2b shows SEM images of the Si mold with a line/space pattern, while Figure 2c presents SEM images of the master mold after spin-coating with four different PMMA solutions, each varying by 0.5 wt%. The spin-coating was performed at a fixed rotational speed of 5000 rpm for 23 s. The results indicate that at PMMA concentrations of 2.5 wt% and 3 wt%, only the space regions of the mold were filled with the solution, while the raised line regions remained uncovered. The 2.5 wt% solution formed a layer approximately 75 nm thick, and the 3 wt% solution resulted in a thickness of about 105 nm. However, at concentrations of 3.5 wt% and 4 wt%, both the space and line regions were covered by the PMMA solution, resulting in PMMA layers of 45 nm and 65 nm thickness, respectively, on the Si lines. When a solution concentration of 2.5 wt% or lower is used for spin-coating followed by the replication process, it is not possible to detach the PMMA pattern from the mold using an adhesive film, as shown in Figure S2. This is likely due to inadequate contact between the adhesive material and the PMMA in the trench or space regions. On the other hand, PMMA patterns obtained from solutions with concentrations of 4 wt% or higher result in a continuous film, where the PMMA in both the line and space regions of the Si mold is interconnected, rather than forming discrete line patterns (see Figure S3 in the Supplementary Materials). This demonstrates that a concentration of 3 wt% is optimal, as it ensures sufficient coverage in the space regions to form replicas while leaving the line regions uncovered.

### 3.3. The Effect of Spin-Coating Rotational Speed on PMMA Film Thickness

**Figure 3:**
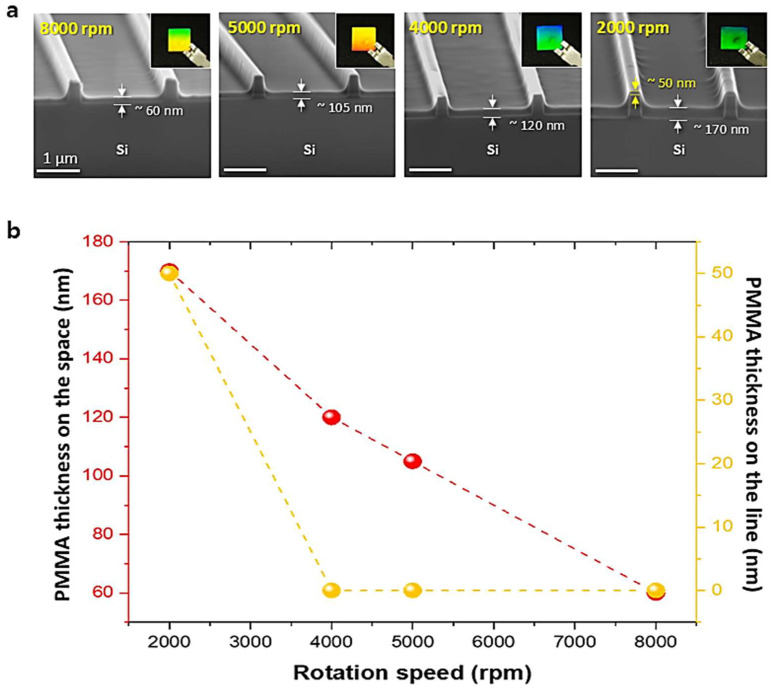
The effect of spin-coating rotational speed on the thickness of the PMMA film. (**a**) Tilted SEM images showing PMMA thin films on the Si line/space mold at different spin-coating speeds. Scale bar: 1 µm. (**b**) Graph illustrating the thickness of the coated PMMA film on the Si mold with lines and spaces, as a function of rotational speed. As the spin-coating speed increases, the thickness of the PMMA film decreases.

At a fixed weight percentage, the spin-coating rotational speed significantly influences the thickness of the PMMA film, and under the appropriate conditions, a discrete pattern can be effectively formed. Figure 3 demonstrates how the amount of solution filled into the mold can be controlled by adjusting the rotational speed during the spin-coating process. Figure 3a shows that as the spin-coating speed decreases, the amount of solution deposited increases. At this point, the concentration of PMMA was fixed at 3 wt%. At 8000 rpm, no PMMA buildup was observed on the line regions, while the space regions were covered with a PMMA layer of approximately 60 nm thickness. At 5000 rpm and 4000 rpm, the line regions remained uncovered, and the space regions were covered with layers of about 105 nm and 120 nm thickness, respectively. However, at 2000 rpm, both the line and space regions were covered by the PMMA solution, with a thickness of about 50 nm on the lines and 170 nm in the spaces. As the rotational speed increased, the amount of solution deposited decreased, and the line regions remained uncovered. Figure 3b illustrates the relationship between spin-coating speed (ranging from 2000 rpm to 8000 rpm) and the thickness of the solution deposited on the line and space regions. An inverse relationship is observed, where the amount of solution on the mold increases as the rotational speed decreases. As a result, at 2000 rpm, the conditions for forming a discrete pattern, where PMMA accumulates only in the space regions, were not met. At 4000, 5000, and 8000 rpm, the conditions for forming a discrete pattern were achieved; however, at 8000 rpm, insufficient PMMA coverage prevented adequate replica formation, creating challenges in proceeding with the nTP process. Based on these results, it can be concluded that a spin-coating speed between 4000 and 5000 rpm is optimal for forming a discrete pattern.

### 3.4. Formation of PMMA Line Pattern on the Target Substrate via nTP Process

**Figure 4:**
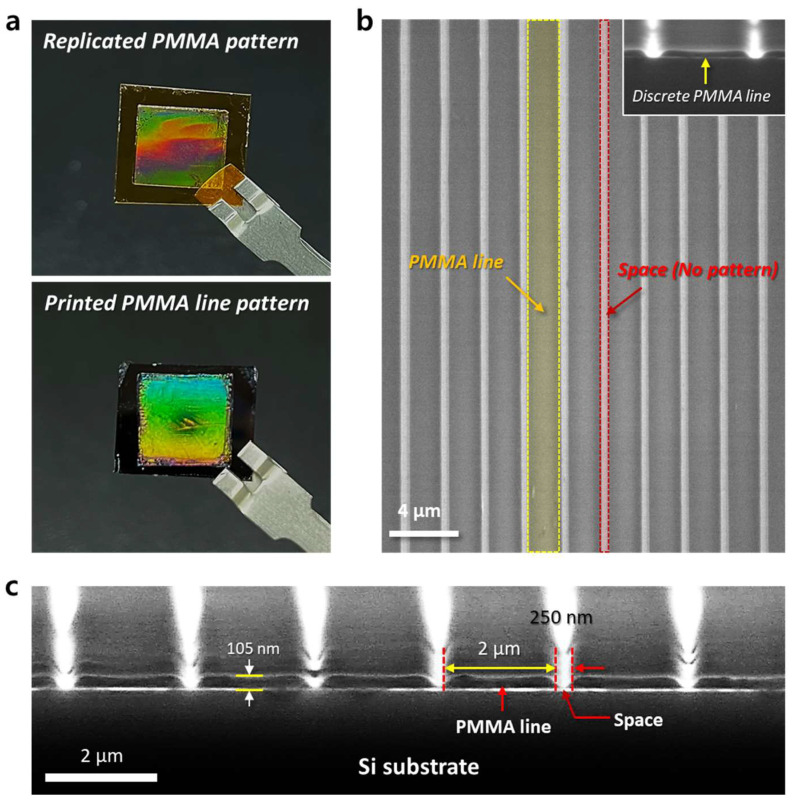
Well-defined, discrete PMMA line patterns after the nTP process. (**a**) Photographs of the replicated PMMA line pattern from the Si mold (upper) and the transfer-printed PMMA line pattern on a planar Si substrate (lower). (**b**) Top-view SEM image showing the periodic PMMA line/space pattern (3.0 wt% solution) transferred at a spin speed of 5000 rpm. Scale bar: 4 µm. (**c**) Tilted SEM image of the discrete PMMA line pattern with a 2 µm width, tilted from the top-view shown in (**b**). Scale bar: 2 µm. The SEM images highlight the well-defined, discrete PMMA line array, with a thickness of approximately 105 nm.

After replicating the PMMA line patterns formed within the spaces of the Si master mold, the next step involves transfer printing them onto the target substrate to create the final discrete PMMA line patterns on the Si wafer. The PMMA concentration was set to 3 wt% and the rotation speed to 5000 rpm, based on the results shown in Figure 2 and Figure 3. The polymer pattern on the mold was replicated using adhesive PI tape and transferred to the Si wafer, as shown in Figure 4a. Figure 4b shows a top-view SEM image of the discrete PMMA line pattern after the transfer printing process, showing the well-defined PMMA pattern. Figure 4c presents a cross-sectional SEM image of the PMMA line/space structure from Figure 4b, clearly showing the distinct features. The transferred PMMA lines exhibit a line width of 2 µm, a space width of 250 nm, and a height of approximately 105 nm. Here, it is essential to emphasize that achieving discrete PMMA line patterns depends on carefully controlling the PMMA weight percentage, spin-coating speed, and duration. This process also demonstrates the capability of the nTP method to successfully pattern PMMA nanostructures on the target substrate. Additionally, we expect that this replication process, based on controlled film thickness, could enable precise patterning across various materials, including polystyrene, poly(3,4-ethylenedioxythiophene) (PEDOT-PSS), quantum dots, and lift-off masks, thereby expanding the potential applications of this method.

### 3.5. 3D PMMA Cross-Bar Structures on Various Surfaces Using Multiple nTP Processes

**Figure 5:**
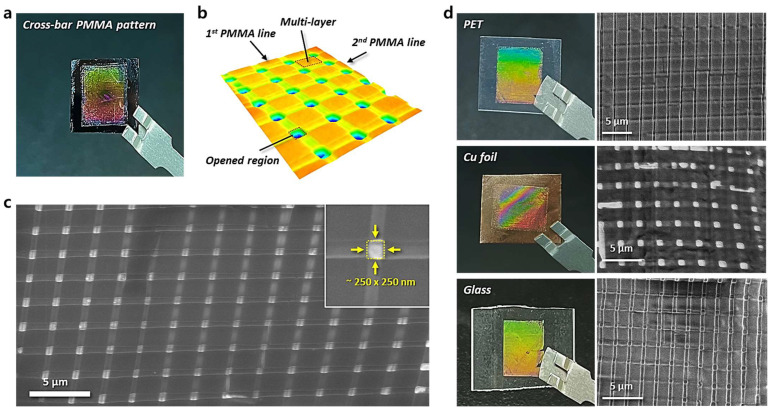
Multi-layer stacking of well-ordered PMMA line patterns on various substrates by the repeated nTP process. PMMA cross-bar patterns were successfully transfer-printed onto several substrates, including (**a**) Si wafer, (**d**) PET, copper foil, and glass. (**a**) PMMA cross-bar structure stacked on a Si wafer using the nTP process. (**b**) Surface plot of a 3D image of the PMMA cross-bar structure, clearly showing the well-organized nanoporous pattern with open areas. (**c**) SEM image of the multi-layered PMMA pattern across a large area. (**d**) Photographs (left) and SEM images (right) of PMMA cross-bar patterns, showing well-defined PMMA nanoporous structures on PET, copper foil, and glass.

Nano-to-microscale structures with a large specific surface area have a wide range of potential applications. To fabricate these porous polymer patterns, a 3D stacking process capable of repeating non-continuous discrete line patterns is required. To achieve this 3D structure stacking, we employed the nTP process as described earlier. Figure 5 demonstrates the formation of 3D PMMA cross-bar arrays on various substrates by repeatedly stacking discrete patterns. Figure 5a–c show the highly ordered porous PMMA cross-bar structure on the Si substrate, achieved through the repeated nTP process. The PMMA line patterns were transfer-stacked at a 90-degree angle to each other, resulting in a cross-bar array. The single-layer regions have a thickness of 105 nm, as shown in Figure 4c, while the double-stacked areas reach approximately 210 nm, highlighting the structural precision achieved in the stacking process. Figure 5b is a 3D surface plot that corresponds to Figure 5c. It clearly shows the open areas, or holes, with dimensions of approximately 250 × 250 nm, where no polymer is present. It can be observed that the areas printed once appear shallower compared to the multi-layered regions, while the areas printed twice are the thickest. Figure 5d presents photographs (left) and SEM images (right) of the PMMA cross-bar patterns, showing well-defined PMMA nanoporous structures on PET, copper foil, and glass. These images confirm the successful transfer and structural integrity of the PMMA cross-bar patterns across different substrate materials, demonstrating the versatility of the nTP process.

## 4. Conclusions

In summary, we demonstrated a simple and effective nanopatterning method to fabricate 3D PMMA cross-bar patterns by stacking discrete periodic line patterns. Key process steps included controlling the polymer solution concentration and adjusting the spin-coating rotation speed, which enabled the successful implementation of the T-nTP process and the formation of discrete PMMA line patterns. By finely tuning the PMMA film thickness and optimizing the weight percentage and spin-coating conditions, we were able to obtain discrete PMMA line structures through the replication and transfer printing process. These periodic 105 nm thick PMMA line patterns were then stacked at a 90-degree angle, producing a porous 3D cross-bar structure with well-defined square-arrayed holes using the T-nTP method. Moreover, the stacking nTP process was demonstrated on various substrates, including planar Si wafers, flexible PET substrates, metallic copper foil, and transparent glass, highlighting the versatility of this method across different materials. The formation of these 3D nanoporous mesh structures is groundbreaking, as it was achieved by directly transfer-printing the replica pattern itself without the need for depositing functional materials. This approach shows great potential for applications requiring a high surface area. We also anticipate that this 3D polymer cross-bar patterning technique can be applied to smart nano-to-microscale fabrication for a range of device applications, including memories, energy harvesters, catalysts, and sensors, all of which benefit from nanostructured architectures.

## Data Availability

The data presented in this study are available on request from the corresponding author.

## Supplementary Materials

The following supporting information can be downloaded at: https://www.mdpi.com/article/10.3390/ma17225609/s1, Figure S1: Schematic of the typical nTP process sequence for spin-coated replica material without functional material coating; Figure S2: Resulting images after the replication process using 2.5 wt% PMMA coating; Figure S3: SEM image of the transfer-printed pattern after the replication process using 4.0 wt% PMMA.
