# Supplementary material for: Controlled Formation of Porous Cross-Bar Arrays Using Nano-Transfer Printing
Source: Materials (Basel). 2024 Nov 16;17(22):5609. doi: 10.3390/ma17225609 (PMC11595979; doi:10.3390/ma17225609)
Supplement: Supplementary file 1 [file materials-17-05609-s001.zip › materials-3286766-supplementary.pdf]

materials

MDPI

Supplementary Materials

Article

Controlled Formation of Porous Cross-bar Arrays using Nano-transfer Printing

Yu Na Kim[1], Eun Bin Kang[1], Tae Wan Park[1], and Woon Ik Park[1]*

[1]Department of Materials Science and Engineering, Pukyong National University (PKNU), Busan 48513, Republic of Korea; kyn510000@gmail.com (Y.N.K), beiunn803@gmail.com (E.B.K) , twpark0125@gmail.com (T.W.P)

* Correspondence: thane0428@pknu.ac.kr (W.I.P); Tel.: +82-51-629-6355

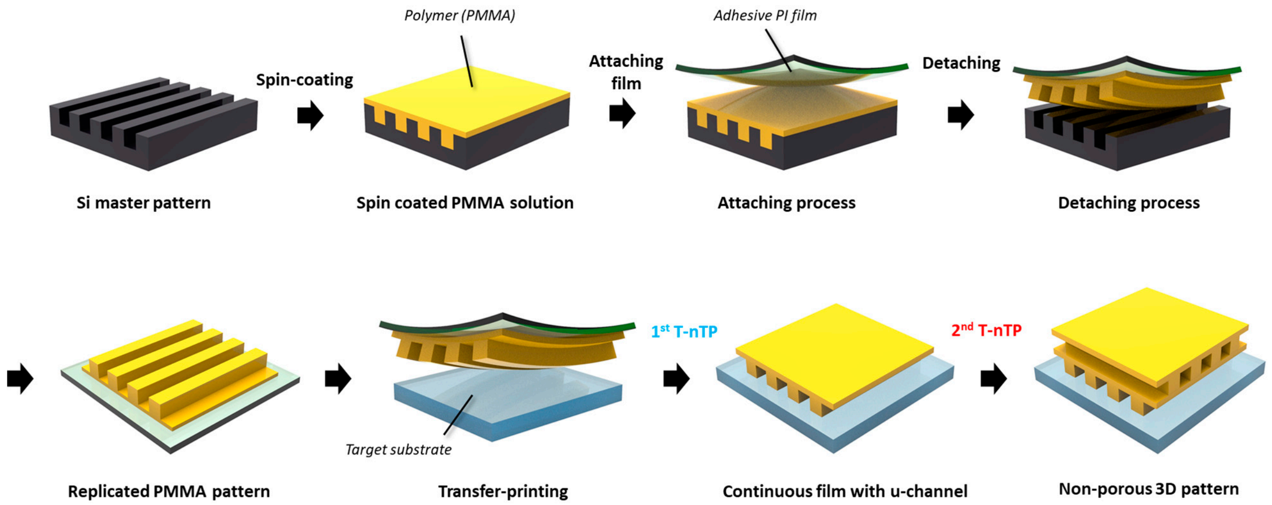

Figure S1. Schematic of the typical nTP process sequence for spin-coated replica material without functional material coating. When the selective filling of PMMA in the mold trenches is not applied, a PMMA film with micro-channels is formed, which prevents the creation of a porous structure in cases of multilayer stacking.

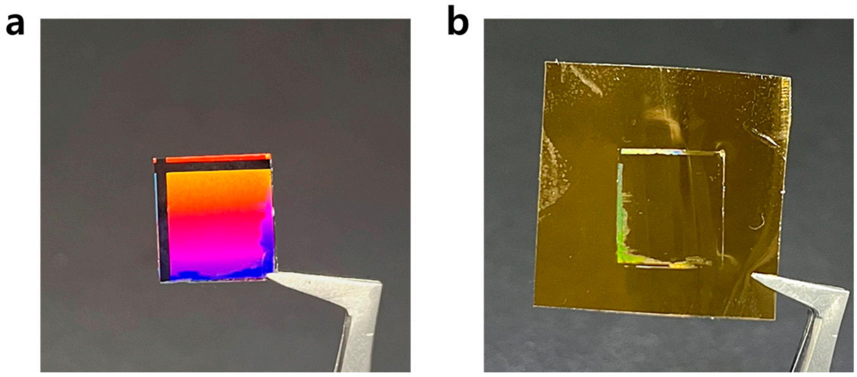

Figure S2. Resulting images after the replication process using 2.5 wt% PMMA coating. (a) Photo image after PMMA spin-coating, (b) Image after replication with an adhesive film. Due to the insufficient thickness of the PMMA film on the trench regions, no replica pattern is obtained, making transfer printing infeasible.

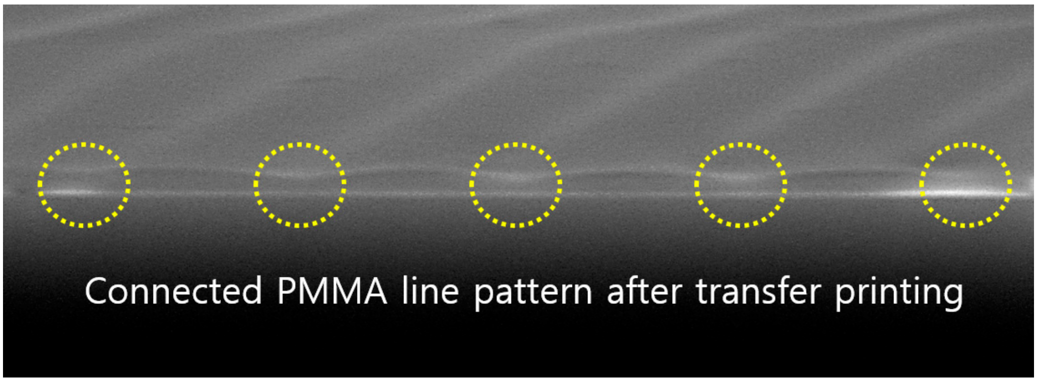

Figure S3. SEM image of the transfer-printed pattern after the replication process using 4.0 wt% PMMA. The PMMA coated on the mesa results in a continuous PMMA line pattern, forming a final film structure.
